# Grand Rounds: Latex-Induced Occupational Asthma in a Surgical Pathologist

**DOI:** 10.1289/ehp.7830

**Published:** 2005-03-31

**Authors:** Judith Green-McKenzie, Debra Hudes

**Affiliations:** University of Pennsylvania Medical Center, Division of Occupational and Environmental Medicine, Philadelphia, Pennsylvania, USA

**Keywords:** formaldehyde, health care worker, latex allergy, occupational asthma, pathology, xylene

## Abstract

Context: Latex allergy and sensitization have been an important problem facing health care workers. Providing a latex-safe environment is the intervention of choice.

Case Presentation: A 46-year-old surgical pathologist presented with increasing shortness of breath for the previous 4 years. Twenty years before presentation, he noted a pruritic, erythematous rash on his hands, associated with latex glove use. Fourteen years before presentation, during pathology residency, he developed a nonproductive cough, wheezing, and an urticarial rash, temporally associated with use of powdered latex gloves. These symptoms improved while away from work. At presentation, he had one-flight dyspnea. His skin prick test was positive for latex, and pulmonary function testing showed mild obstruction, which was reversible with bronchodilator use. Because the patient was at risk for worsening pulmonary function and possible anaphylaxis with continued exposure, he was removed from the workplace because no reasonable accommodation was made for him at that time.

Discussion: The patient’s presentation is consistent with latex-induced occupational asthma. Initially noting dermal manifestations, consistent with an allergic contact dermatitis secondary to accelerators present in latex gloves, he later developed urticaria, flushing, and respiratory symptoms, consistent with a type I hypersensitivity reaction to latex. He also has reversible airways disease, with significant improvement of peak expiratory flow rate and symptoms when away from work.

Relevance to Clinical or Professional Practice: The ideal treatment for latex sensitization is removal from and avoidance of exposure. Clinicians should consider occupational asthma when patients present with new-onset asthma or asthmatic symptoms that worsen at work.

## Case Presentation

A 46-year-old male surgical pathologist presented to our clinic complaining of a 4-year history of increasing shortness of breath. He had been in good health until 20 years prior while in medical school, when he noted a pruritic, erythematous rash on the dorsal aspect of his hands whenever he wore latex gloves. He often applied steroid cream to the rash, but it usually did not resolve unless he refrained from using latex gloves. This rash, associated with latex glove use, persisted during his internal medicine residency. Approximately 14 years before presentation, at the beginning of his pathology residency, he noted that the rash involved his arms. He developed an episodic, nonproductive cough, wheezing, and occasional chest tightness, which occurred at work when he used powdered latex gloves. These symptoms were mild and did not interfere with his vigorous exercise program. He did not seek medical attention.

After completing his residency, the patient worked as a hospital-based surgical pathologist. Typical daily activities involved cutting tissue and frozen sections and preparing slides. He changed gloves several times each day. He did reasonably well until 4 years before presentation (1993), when his symptoms worsened. He then experienced cough and dyspnea within 30 min of starting work. These symptoms, which continued throughout the workday and improved once he left work, seemed especially severe on the first day of the workweek and worsened as the week progressed. The use of xylene and formaldehyde exacerbated his symptoms. He noted an intermittent rash on his upper extremities and torso, occasional flushing with exposure to latex, postnasal drip, progressive dyspnea on exertion, and dyspnea and coughing when he laughed. He noted heavy breathing if he “flipped” his gloves off, and he described an episode of “passing out” 1 year earlier when he “flipped” his gloves off and placed his hands over his mouth and nose. He was taken to a local emergency department, where he was diagnosed as having had a vasovagal episode. He was returned to work without intervention.

The patient’s wife and co-workers started commenting on his cough, noting that he “breathed heavily.” He became self-conscious about his cough and about constantly having to clear his throat. There was no seasonal variation to his symptoms. The patient attempted to reduce his exposure to powdered natural rubber latex (NRL) gloves, formaldehyde, and xylene. For example, he switched to non-powdered latex gloves, although his co-workers continued to use the powdered form. He replaced eyecups on the microscope once he realized that they contained latex. He instructed his staff to allow an hour for drying slides fixed with formaldehyde and xylene before sending them to him to be read. His symptoms persisted, however, prompting him to seek medical attention.

The patient subsequently consulted with an allergist, an otorhinolaryngologist, and a dermatologist. Skin biopsy of his rash revealed changes consistent with acute urticaria. Latex skin prick tests were positive to latex glove extracts. Skin prick tests were positive to dust, cat dander, and mold antigens, and a computerized tomography (CT) scan of the sinuses revealed nasal polyps in the maxillary sinus. He was diagnosed with chronic sinusitis, asthma, and allergic rhinitis. Treatment included antibiotics and a steroid taper. The patient was started on Serevent (GlaxoSmithKline, Research Triangle Park, NC), Flovent (GlaxoSmithKline), and Proventil (Schering, Kenilworth, NJ) inhalers and returned to work with the recommendation that he use a surgical mask while at work. His symptoms continued to progress, and he presented to us 2 months later, by which time he was experiencing single-flight dyspnea.

The patient’s past medical history was remarkable for hypertension, nasal polyps, and near syncope. He denied any previous diagnosis of asthma, allergy, hives, or anaphylaxis. His family history was remarkable for asthma in a sister and a paternal uncle. He denied use of alcohol, cigarettes, or illegal drugs and denied allergies to medications or environmental substances. He gave a history of chest tightness when he ate fruit such as banana, avocado, and kiwi. His occupational history was remarkable for work in the medical field (Table 1). On physical examination, he was a well-nourished, well-developed white male in no acute distress whose vital signs were within normal limits. His examination was remarkable for a body mass index of 30, hyperemic conjunctivae, boggy nasal mucosa, an erythematous urticarial rash on his right shoulder, and diffuse expiratory wheezing.

**Figure 1:**
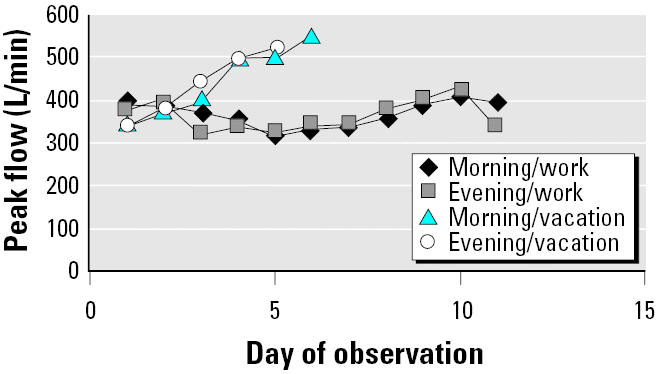
The patient’s morning and evening PEFRs recorded in 1997 on 11 consecutive days while at work (Sunday, 2 November, through Wednesday, 12 November) and on 6 consecutive days while on vacation (Wednesday, 13 November, through Tuesday, 18 November).

Laboratory evaluation revealed a normal electrocardiogram. Chest X ray showed poor inspiration; CT of the chest showed mild bronchial wall thickening consistent with mild airways disease; pulmonary function tests (PFTs) were remarkable for mild obstruction with acute bronchodilator response (Table 2); and a radioallergoimmuno-absorbent assay (RAST) test for latex IgE antibody was negative. His peak expiratory flow rate (PEFR) diary during an 11-day work period and a subsequent 6-day vacation period showed significant improvement (20% in the morning, 22% in the evening) while he was away from work (Table 3) and progressive improvement during successive days of vacation (Figure 1).

The provision of a latex-safe environment was explored with hospital administration and deemed not feasible at that time. A full-face dual-cartridge respirator was recommended and tried in consultation with a certified industrial hygienist. However, it interfered with the patient’s ability to communicate, and he was unable to tolerate wearing it for an 8-hr day. We felt that he was at risk for potentially fatal anaphylaxis, as well as irreversible and impending structural damage to his lungs, given his long history of exposure and disease severity. In order to eliminate exposure to NRL, the patient was removed from the work-place. He was advised to avoid contact with latex, carry injectable epinephrine, and wear a MedicAlert bracelet (MedicAlert Foundation International, Turlock, CA). Despite removal from the workplace shortly after presentation, the patient’s pulmonary status did not improve. He is maintained on steroids and immunosuppressive agents and has not been able to return to work as a surgical pathologist.

## Discussion

### Latex allergy and sensitization.

The use of powdered high-protein NRL gloves is recognized as the major environmental risk factor for latex sensitization and allergy in the health care field (Levy et al. 1999; Wild and Lopez 2003). The widespread use of NRL gloves in the health care industry started in the 1980s as health care facilities complied with Universal Precautions [Occupational Safety and Health Administration (OSHA) 1991]. After the first report of a case of immediate hypersensitivity to NRL (Nutter 1979), NRL allergy became increasingly recognized as a problem among health care workers (Garabrant and Schweitzer 2002). NRL, used in the production of latex gloves, is derived from the milky sap of the commercial rubber tree, *Hevea brasiliensi* (Atkins 1999). The sap of this tree is a complex mixture of protein, lipid, and phospholipid. The protein content varies depending on country of harvest location, environmental conditions, and manufacturing process. Sixty of the 240 proteins in NRL have been found to be allergenic (Levy et al. 1999).

Freshly harvested latex is treated with ammonia and other preservatives to prevent its deterioration during transport to factories; it is then treated with antioxidants and accelerators before being shaped into the final product. Increased washing time in glove manufacture can lead to a decrease in the amount of soluble protein in the final product (Yunginger et al. 1994), hence decreasing the antigenicity of the glove. The product is frequently dry-lubricated with cornstarch or talc powder to improve ease of donning the glove. Latex allergen elutes onto the powder, providing a source for respiratory exposure (Yunginger et al. 1994). Notably, synthetic rubber elastomers (butyl rubber, polymers of 2-chlorobutadiene, co-polymers of butadiene and acrylonitrile) do not cause or contribute to allergic sensitization; people who are sensitized to NRL proteins can safely use products made from synthetic rubbers (OSHA 1999; Renaud 1993).

Most reactions associated with NRL can be classified into three main categories: irritant contact dermatitis (ICD), allergic contact dermatitis (ACD), and an immediate hypersensitivity reaction (Felt-Ahmed et al. 2003). ICD is confined to the skin and occurs when the skin has direct contact with the glove. ICD represents a type of contact dermatitis and is not allergic in nature. The second type of reaction, ACD, is a delayed hypersensitivity reaction (type IV) thought to be a result of exposure to the accelerators, which can lead to the activation and release of lymphokines by sensitized T lymphocytes rather than to the latex itself (Atkins 1999). Endotoxins, which may be present as contaminants, have also been implicated as causing ACD (Charous et al. 1997). Features of ACD are pruritic rash, local erythema, swelling, blistering, weeping, and crusting. These symptoms generally occur 1–2 days after exposure but also may occur from several hours to several days postexposure (Felt-Ahmed et al. 2003).

The third type of reaction, the type I, immediate-type hypersensitivity reaction, relies on previous sensitization of the immune system to latex antigens and to the generation of IgE antibodies directed specifically at latex proteins and is the most serious of the three (Atkins 1999; Vandenplas et al. 1995). Signs and symptoms include asthma, rhinitis, conjunctivitis, generalized urticaria, and mucous membrane swelling. Anaphylaxis, the most dreaded complication, may also occur in a sensitized patient and has been recorded to have occurred as a result of donning gloves, being in the presence of others who have put on gloves, during surgery, and during dental and medical examinations (Vandenplas et al. 1995). In 1991, a latex barium enema tip associated with 16 deaths was recalled by the Food and Drug Administration (FDA); this led to an increased awareness of the risk of life-threatening type I allergy associated with NRL devices (Gelfand 1991). Sensitization occurs after multiple exposures over a highly variable time, the latency period ranging from several weeks to as long as 30 years (Malo et al. 1992). Once sensitization occurs, there is considerable variability in the type and severity of allergic symptoms, occurring from within 30 min (anaphylaxis, angioedema) to more than hours and days after exposure. Asthma symptoms are highly variable in their onset, duration, and intensity, the more severe cases being associated with multiple and prolonged exposures occurring over many months to years (Felt-Ahmed et al. 2003).

The prevalence of latex sensitization has been estimated to be between 5 and 17% in health care workers (Malo et al. 1992), versus between 5 and 10% in the general population (Felt-Ahmed et al. 2003). The factors associated with an increase in the risk of latex sensitization among health care workers include the duration of exposure and the intensity of exposure to NRL gloves. Intensity of exposure is measured by the number of pairs of gloves used per day and the amount of powdered glove use (Garabrant and Schweitzer 2002). The mechanical and irritant reaction to the powder may lead to a breakdown of the skin barrier, further enhancing exposure to the latex protein (Levy et al. 1999). In addition, the powder disseminates into the environment, carrying the latex protein with it, providing a respiratory route of exposure (Baur et al. 1993). An increase in latex sensitization is seen with particular jobs and departments in health care probably as a result of a relatively higher exposure to NRL gloves. Laboratory workers have been found to have the highest incidence of latex sensitization, 4% per year, whereas the incidence of latex sensitization among health care workers in general has been estimated at 1–2.5% per year; pathology staff has been found to have a 14% prevalence of latex sensitization (Garabrant and Schweitzer 2002).

Atopic individuals are more easily sensitized to allergens and, as such, are at greater risk of developing a latex allergy than are individuals who are not atopic (Felt-Ahmed et al. 2003). Atopy is a hypersensitivity state or allergy with hereditary predisposition. Atopic individuals may have a personal or family history of eczema, asthma, or hay fever or a tendency to develop specific IgE antibodies after exposure to common environmental substances, although many do not. The tendency to develop some form of allergy is inherited, but the specific clinical form, such as hay fever, asthma, or eczema, is not (Wild and Lopez 2003). Skin tests to common environmental allergens such as pollen, animal dander, molds, and house dust mites are used to evaluate atopic status. One looks for the immediate IgE-mediated wheal and flare reaction. Clinical associations have been reported between latex allergy and allergy to several fruits and vegetables, such as avocado, kiwi fruit, banana, potato, tomato, chestnut, and papaya (Beezhold et al. 1996). Several latex allergens (e.g., Heb b2, 5, 6.02, and 7) have varying degrees of amino acid sequence homology with allergens in seed-producing plants (Wagner and Breiteneder 2002). Some patients report that food allergy preceded the latex allergy, and others report the converse (Beezhold et al. 1996).

Sensitization can be documented by the use of a skin prick test using extracts prepared from suspected substances, such as latex, in the work environment. Detection of specific IgE antibodies suggests a cause-and-effect relationship. Licensed extracts of latex for skin testing, available in Europe, have been found to be safe and reliable for detecting latex-specific IgE. The United States does not have licensed commercial latex extracts. As a result, skin testing is done with unstandardized office-prepared latex extracts, which vary widely in allergen content (Ownby 2003). Specific IgE antibodies can also be studied *in vitro* using a blood test, the RAST assay (Wild and Lopez 2003). Tests for latex-specific IgE such as the RAST are less sensitive and specific than are skin prick tests, with sensitivity ranging between 73 and 80% and specificity ranging between 90 and 97% (Ownby 2003). The laboratory to which this patient’s RAST was sent reports a 30% false-negative rate (Hamilton 1999).

### Latex-induced occupational asthma.

Occupational asthma (OA) can be defined as the presence of variable airflow obstruction and bronchial hyperresponsiveness caused by a substance found in the workplace (Tilles and Jerath-Tatum 2003). OA differs from preexisting asthma, which is exacerbated by exposure to agents in the workplace (Wild and Lopez 2003). However, OA may occur in conjunction with preexisting asthma, because OA involves the new onset of sensitization to a workplace antigen or allergen with the development of respiratory disease. A person with preexisting asthma and allergies may develop OA to a workplace allergen. Another feature of OA is the occurrence of nasal, ocular, or contact urticarial symptoms that precede asthma symptoms. The presence of these symptoms is helpful, but not necessary, in establishing the diagnosis.

Other features include the association of prolonged exposure with worsening asthma symptoms at work, the development of more pervasive symptoms while at work, and the presence of a latency period between the initial exposures to the inciting agent where symptoms may develop from weeks to > 20 years after exposure (Chan-Yeung 1987; Tilles and Jerath-Tatum 2003; Wild and Lopez 2003). Reactive airways dysfunction syndrome (RADS) is a form of OA that does not require a latency period. RADS can occur acutely, within 24 hr, after one single exposure to an irritant (Tilles and Jerath-Tatum 2003). OA symptoms may resolve in some individuals, whereas others remain symptomatic for years. Approximately 10% of adult asthma cases are attributed to an occupational etiology (Blanc and Toren 1999). More than 250 agents encountered in the workplace have been shown to induce asthma in susceptible individuals (Wild and Lopez 2003).

Atopic individuals are at greater risk of developing OA, especially when working in an industry where high-molecular-weight proteins such as latex proteins are present. Other high-molecular-weight proteins known to cause OA are flour and animal antigens (Wild and Lopez 2003). Allergic OA is seen in individuals who develop sensitization to a specific chemical agent in the workplace. Persons with allergic OA tend to develop bronchospasm and airway inflammation upon exposure, even to low concentrations of the specific workplace agent to which they are sensitized (Paggiaro et al. 1994). NRL-induced OA, an IgE-mediated process, is initiated when the allergen-bearing particles deposit onto the mucosal surfaces of the respiratory tract. Of the health care workers estimated to be sensitized to latex, 41–69% of them are estimated to have respiratory symptoms with exposure (Lagier et al. 1992).

Various criteria are used in making the diagnosis of OA. A significant postbronchodilator response is considered to have occurred if PFTs demonstrate an increase in forced vital capacity (FVC) or forced expiratory volume in 1 sec (FEV_1_) of 12% above baseline and an absolute change of 0.2 L (American Thoracic Society 1991). Methacholine challenge testing, the gold standard for establishing the diagnosis of asthma, can also be used to show non-specific bronchial hyperreactivity. An abnormal test result is defined by the concentration of methacholine that drops the baseline FEV_1_ by 20% (Tan and Spector 2003). Medical and work histories may be used to help ascertain a temporal association between the patient’s symptoms and work, as well as to rule out other causes for the symptoms.

One recommendation for confirming the diagnosis of OA, using pre- and postshift spirometry or PEFR, is by showing a significantly decreased obstructive pattern at work compared with being away from work. For example, the PEFR should be measured approximately every 2–3 hr during a 2-week period at work and during a 1–2 week period away from work. OA is confirmed by finding a ≥20% reduction in PEFR at work versus away from work or by finding at least a 20% diurnal variability of mean work PEFR, with the disappearance of this variability when away from work (Tilles and Jerath-Tatum 2003). PFTs are most useful in suggesting an occupational cause for asthma when they show a decrease in FEV_1_ of at least 15% when comparing results obtained before and after a period of work (Greaves 2003). The diagnosis of OA is usually confirmed by a combination of findings. The history and physical exam should be consistent with this diagnosis; spirometry or methacholine challenge testing should demonstrate variable airflow obstruction; and serial peak flows should confirm that bronchial hyperreactivity is triggered by work-place exposures to specific agents.

### Role of formaldehyde and xylene.

Formaldehyde is an upper respiratory tract irritant, exacerbating bronchial airflow obstruction or hyperreactivity. It can exacerbate asthma and precipitate wheezing in those with underlying asthma or bronchial hyperreactivity. Formaldehyde may cause an immune response by forming a hapten, a complex of a protein and a low-molecular-weight compound, which can induce an IgE response, although this is uncommon (Rutchik 1999). Xylene, an aromatic hydrocarbon used in medical technology as a solvent and fixative, may exacerbate asthma and rhinitis. Other agents to which our patient may have been exposed during his daily work as a pathologist that he did not identify as specific triggers to his symptoms—but that are associated with respiratory and dermatologic symptoms—are glutaraldehyde, phenol, and ethylene glycol (Rutchik 1999).

### Treatment and workplace accommodation.

Disability from occupationally induced allergies is compensable under Workers’ Compensation law (Phillips et al. 1999). A worker with OA or NRL-induced anaphylaxis is considered to be 100% impaired from performing his or her specific job if the job entails exposure to the causative agent (American Thoracic Society 1993; Bernstein 2002). Under the Americans with Disabilities Act (1990), reasonable work-place accommodation must be made to allow a disabled worker to perform the “essential functions” of the job. The ideal treatment for latex sensitization is prevention of exposure, best achieved by identifying and removing all latex-containing products in the workplace. Latex aeroallergen levels are significantly reduced when medical centers eliminate powdered NRL gloves from the work environment, replacing them with nonpowdered synthetic rubber gloves (Swanson et al. 1994). This workplace modification has been found to be most effective and is associated with an improvement in respiratory and dermatologic symptoms in health care workers and with a reduction in the number of new cases of latex sensitization and allergy (Bernstein et al. 2003; Hunt et al. 2002; Saary et al. 2002; Swanson et al. 1994). It has also been shown to be cost-effective, considering the cost incurred by disability from latex allergy and asthma (Allmers et al. 2002; Phillips et al. 1999).

Many medical devices and products, as well as many common household and everyday items, contain NRL. However, identifying latex-containing products was made simpler when the FDA mandated that all NRL-containing medical devices be labeled as such and that health care sites provide non-latex-containing alternatives (FDA 1997). The FDA concluded that this intervention is affordable for manufacturers (FDA 1997). Extensive lists of NRL-containing products and latex-safe alternatives are also available (Spina Bifida Association of America 2004). Despite this, however, it is difficult to render and maintain an environment completely latex-free. Furthermore, NRL-containing items may also be inadvertently brought into an area. As a result, “latex-safe” is the preferred term.

Prevention of exposure may also be carried out through engineering and industrial hygiene controls and through the use of personal protective equipment. Latex aeroallergen levels may be monitored, and engineering controls can include exhaust ventilation equipment (Reiter 2002), although the use of a laminar flow glove-changing station has not been shown to reduce latex aeroallergens (Swanson et al. 1994). Work-practice controls, such as cleaning the area, might help to eliminate or minimize the hazard. Environmental controls such as HEPA-filtered vacuuming and wet wiping of surfaces with isopropyl alcohol may reduce latex allergen on surfaces (Reiter 2002).

The worker may also use personal protective equipment such as a respirator. Respirators can provide additional protection and mitigate the hazard but are not the method of choice for controlling exposures. There are various categories of respirators. Air-purifying respirators may use negative pressure (the user pulls air through the respirator), or air is supplied through a powered source (powered air-purifying respirator). They remove much of the toxicant from the inhaled air by filtration, adsorption, or absorption. Atmosphere-supplying respirators, such as the self-contained breathing apparatus (supplies air from a source such as a tank carried by the user), and the airline respirator (uses air supplied via a hose from a distant source), provide air from an independent source as opposed to purifying ambient air.

Most respirators require a tight seal between the mask and the user’s face, although some are loose fitting. Masks are quarter, half, or full face depending on the portion of the face that is covered [Harber et al. 2005; National Institute for Occupational Safety and Health (NIOSH) 2005]. Laminar flow HEPA-filtered helmets have been found to be effective in reducing the symptoms of latex-induced asthma, rhinitis, and conjunctivitis (Laoprasert et al. 1998). Respirators may interfere with vision, hearing, mobility, ability to communicate, and the use of tools such as stethoscopes and microscopes. They may be uncomfortably warm, with tight-fitting head straps, and may also lead to increases in resistance to breathing, dead space, and physical load. These factors, among others, may contribute to a functional inability to keep the respirator on for more than a brief period of time in some persons. Recommendations of a certified industrial hygienist should be used when available (Harber et al. 2005; NIOSH 2005).

Sensitized workers with severe asthma and other life-threatening allergic reactions must be removed from the workplace if exposure cannot be prevented, because the asthmatic response can occur at minute levels of exposure (Ehrlich 1994). Although not documented in individuals with OA due to NRL, evidence from other sensitizing agents, such as western red cedar asthma and toluene diisocyanate, indicates that repeated exposures to the inciting agent can increase the severity of the asthma, and the disease process may even progress after removal from exposure (Banks et al. 1990; Butcher et al. 1982; Chan-Yeung et al. 1982; Cote et al. 1990). Ultimately, irreversible lung damage and death can result from repeated exposure (Banks et al. 1990; Chan-Yeung 1987).

Removing the employee from the work-place has personal, social, and economic implications. The latex-allergic health care worker may experience psychological distress secondary to coping with the adjustment and may respond with anger, depression, anxiety, and denial. Self-esteem, interpersonal relationships, and economic well-being may be adversely affected when an individual is unable to maintain his current profession with the possible loss of future earnings or forced early retirement. These factors, among others, may lead the health care worker to delay seeking much needed medical attention (Charous et al. 2002a). In addition to eliminating exposure to latex, the treatment for OA is the same as for other types of asthma (Wild and Lopez 2003). Workers with latex sensitization and latex-induced OA should be counseled to wear a MedicAlert bracelet and carry injectable epinephrine with them at all times. They should also be counseled as to what items contain latex and to avoid dermal, mucosal, or serosal contact with them (Howarth 2001).

## Conclusion

This case describes a surgical pathologist whose presentation is consistent with a diagnosis of latex-induced OA. It shows how exposure to a high-molecular-weight protein, latex, led to ACD. Repeated exposure to the inciting agent over a latency period of several years led to latex sensitization and ultimately to latex-induced OA in this atopic indiviual. He did not give a clear history of anaphylaxis, but he was diagnosed with “near syncope” of unknown etiology after flipping his gloves off and placing his hand over his nose and mouth, after which he was returned to work without intervention. Skin prick test, which is diagnostic for the presence of IgE-mediated allergy to latex, was positive to several latex-containing extracts. Although his serum IgE, or RAST, to one type of latex protein was negative, false-negative tests do occur (Hamilton 1999). The patient’s medical and occupational history, in combination with his spirometry and PEFR measurements, supports the diagnosis of OA, reversible airways disease responding to bronchodilators with symptoms that are worse at work and improve away from work. Formaldehyde and xylene probably acted as irritants, exacerbating his pulmonary symptoms.

The mainstay of treatment for latex-induced OA is to prevent contact of the worker with the inciting agents. Creating a latex-safe environment is the provision of choice (Charous et al. 2002b). However, this provision was not made at the time. Given the long period of the patient’s exposure and the severity of his disease, there was concern that his pulmonary function would continue to decline with continued exposure and that he was at risk for anaphylaxis. We thought removal from the workplace was the best way to protect the patient from exposure. Despite removal from inciting agents, the patient’s symptoms and pulmonary status did not improve. He remains out of work and is maintained on steroids and immunosuppressive agents. If his condition been identified and removal from exposure occurred sooner, his disease may not have progressed. Prompt identification of latex allergy and sensitization, as well as reduction or elimination of the hazard, may allow the patient to continue working in his environment and prevent progression of disease. Clinicians should consider OA in patients who present with new-onset asthma or who present with asthma symptoms that worsen during or after work.

## Figures and Tables

**Figure:**
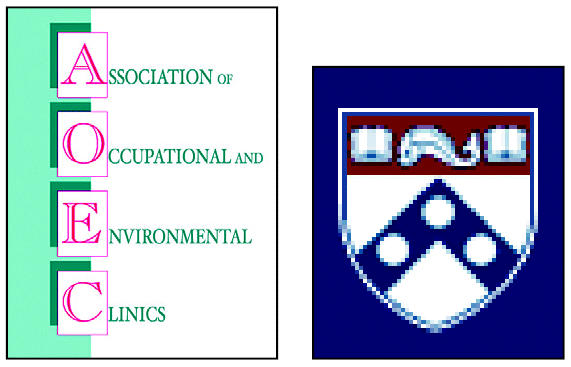
Unversity of Pennsylvania Medical Center

**Table 1:** The chronological relationship between the patient’s occupational exposure and the appearance of symptoms.

Year	Occupation	Symptoms
1977	Medical student	Rash on dorsum of hands with latex glove use; does not clear with steroid use
1979	Internal medicine resident	Continued rash on dorsum of hands with latex glove use
1984	Pathology resident	Rash on hands and arms, urticaria, wheezing, chest tightness, chronic cough
1987	Surgical pathologist	Diagnosed with nasal polyps
1993	Surgical pathologist	Notes dyspnea within 30 min of work and with coughing and laughing
1996	Surgical pathologist	Allergist evaluation results in diagnosis of asthma and allergic rhinitis; emergency department evaluation results in diagnosis of “near syncope” after he flipped off gloves and covered mouth and nose with hands
1997	Surgical pathologist	Presents to our clinic with single flight dyspnea; removed from workplace because no reasonable accommodation made at work

**Table 2:** Spirometry results before and after bronchodilator use showing FEV_1_ and FVC.

	Prebronchodilator	Percent predicted	Postbronchodilator	Percent predicted	Percent change
FEV_1_ (L)	2.65	67	2.98	75	13
FVC (L)	3.96	81	4.47	91	13
FEV_1_/FVC	67	—	67	—	—

Abbreviations: FEV_1_, forced expiratory volume in 1 sec; FVC, forced vital capacity.

**Table 3:** Mean morning and evening PEFRs while at work and during vacation, measured in the morning and in the evening at bedtime both before using asthma medication.

	Mean PEFR		
Time	Work	Vacation	Percent increase
Morning (L/min)	368	443	20
Evening (L/min)	361	441	22
Percent increase	−2	−0.5	—
PEFR range (L/min)	320–425	340–550	—
